# Identification of Critical Elements for Regulation of Inorganic Pyrophosphatase (PPA1) in MCF7 Breast Cancer Cells

**DOI:** 10.1371/journal.pone.0124864

**Published:** 2015-04-29

**Authors:** Dipti Ranjan Mishra, Sanjib Chaudhary, B. Madhu Krishna, Sandip K. Mishra

**Affiliations:** Cancer Biology Laboratory, Gene function and regulation Group, Institute of Life Sciences, Bhubaneswar, Odisha, India; University of Missouri-Kansas City, UNITED STATES

## Abstract

Cytosolic inorganic pyrophosphatase plays an important role in the cellular metabolism by hydrolyzing inorganic pyrophosphate (PPi) formed as a by-product of various metabolic reactions. Inorganic pyrophosphatases are known to be associated with important functions related to the growth and development of various organisms. In humans, the expression of inorganic pyrophosphatase (PPA1) is deregulated in different types of cancer and is involved in the migration and invasion of gastric cancer cells and proliferation of ovarian cancer cells. However, the transcriptional regulation of the gene encoding PPA1 is poorly understood. To gain insights into *PPA1* gene regulation, a 1217 bp of its 5’-flanking region was cloned and analyzed. The 5’-deletion analysis of the promoter revealed a 266 bp proximal promoter region exhibit most of the transcriptional activity and upon sequence analysis, three putative Sp1 binding sites were found to be present in this region. Binding of Sp1 to the *PPA1* promoter was confirmed by Electrophoretic mobility shift assay (EMSA) and Chromatin immunoprecipitation (ChIP) assay. Importance of these binding sites was verified by site-directed mutagenesis and overexpression of Sp1 transactivates *PPA1* promoter activity, upregulates protein expression and increases chromatin accessibility. p300 binds to the *PPA1* promoter and stimulates Sp1 induced promoter activity. Trichostatin A (TSA), a histone deacetylase (HDAC) inhibitor induces *PPA1* promoter activity and protein expression and HAT activity of p300 was important in regulation of PPA1 expression. These results demonstrated that *PPA1 *is positively regulated by Sp1 and p300 coactivates Sp1 induced *PPA1* promoter activity and histone acetylation/deacetylation may contribute to a local chromatin remodeling across the *PPA1* promoter. Further, knockdown of *PPA1* decreased colony formation and viability of MCF7 cells.

## Introduction

Inorganic pyrophosphatase is an enzyme which catalyzes hydrolysis of inorganic pyrophosphate (PPi) molecule into two inorganic phosphates (Pi). PPi is produced in the cell by various metabolic reactions, such as nucleic acid, protein and polysaccharide synthesis and hydrolysis of PPi by PPA1 is thermodynamically favorable to these reactions [1]. PPi level in the cell needs to be regulated as deregulated PPi metabolism has been associated with diseases [2]. Inorganic pyrophosphatase expression was found to be involved in the growth *E*. *Coli* [3] and molting and development of *Ascaris suum* [4]. In *C*. *elgans*, a null mutant of inorganic pyrophosphatase was developmentally arrested at the larval stage with intestinal morphology and functional defects [5]. Defects in inorganic pyrophosphatase were found to be associated with cell cycle arrest and cell death in fermenting yeast [6]. Increased expression and activity of cytosolic inorganic pyrophosphatase was found in rat and mice liver with aging [7, 8]. Inorganic pyrophosphatase is also involved in the neurite growth in mouse neuroblastoma cell line and in rat cortical neurons [9].

In humans, cytosolic inorganic pyrophosphatase (PPA1) was found to be overexpressed in many types of cancer such as, breast cancer [10, 11], lung cancer [12], ovarian cancer [13], hepatocarcinoma [14] and primary colorectal cancer [15]. Altered metabolism is now considered as an emerging hallmark of cancer and cancer cells are thought to maintain their high proliferation rate through the metabolic alterations [16]. Recently, PPA1 was found to be associated with cell migration, invasion [17] and proliferation [18] of cancer cells. However, the mechanism of human *PPA1* gene regulation is not known. In the present study, we have carried out the characterization of the *PPA1* promoter region and tried to get insights into the transcriptional regulation of this gene.

First, a 1217 bp of the 5’- flanking region of the human *PPA1* gene was cloned and analyzed for its promoter activity by serial deletion analysis. Three putative Sp1 binding sites were identified in the *PPA1* minimal promoter region and the binding of Sp1 to the *PPA1* promoter was confirmed by *in-vitro* and *in-vivo* binding assays. The functional importance of the Sp1 binding sites in the *PPA1* gene regulation was demonstrated by site directed mutagenesis and role of Sp1 in the regulation of *PPA1* was studied by luciferase assay, western blot analysis and chromatin accessibility assay. Further, we studied whether any coactivator is involved in the *PPA1* regulation and found that p300 could bind to the *PPA1* promoter to transactivate its activity. Also, p300 can further potentiate Sp1 mediated transactivation of *PPA1* promoter. Probable role of histone acetylation/deacetylation event in the *PPA1* regulation was studied by treating the cells with a specific HDAC inhibitor, TSA and an increase in promoter activity and PPA1 protein expression with the TSA treatment indicate towards a chromatin remodeling event across the *PPA1* promoter. Moreover, knockdown of *PPA1* expression led to reduced colony formation and viability of MCF7 cells.

## Materials and Methods

### Ethics statement

All experimental protocols involving animals used in this study were approved by the Institutional Animal Ethics Committee (IAEC) of Institute of Life Sciences, as per Government of India guidelines.

### Cell culture

The human breast cancer cell line (MCF7) was purchased from NCCS (Pune, India) and cultured in Dulbecco’s Modified Eagle’s Medium (DMEM) supplemented with 10% (v/v) fetal bovine serum (FBS) (PAN Biotech) at 37°C with 5% CO_2_.

### Primer extension analysis

Total RNA was isolated from MCF7 cells using TRI Reagent (Sigma) according to the manufacturer’s protocol. A 21-nucleotide-long primer PPA1PER (5'-agtgccggagtcctgccgcc-3'), which is complementary to the -20 to -1 region of *PPA1* exon-1 (Genbank accession no: NM_021129), was used for primer extension analysis. Briefly, 5′-end-labeled PPA1PER primer was annealed with 50 μg of total RNA at 60°C for 1 h, cooled to room temperature and reverse transcribed at 42°C for 1 h using primer extension system (Promega) according to the manufacturer’s instructions. The same primer was used for the sequencing reactions of cloned *PPA1* promoter containing exon-1. Sequencing reactions and primer extension product were separated side by side on a 7.5% polyacrylamide gel containing 7 M urea, dried, and autoradiographed.

### Nuclear extract preparation

Nuclear extract from MCF7 cells was prepared as per previously [19] with little modification. Briefly, about 1x10^7^ cells were washed two times with PBS and resuspended in 500 μl of Buffer A (10 mM HEPES (pH 7.9), 10 mM KCl, 0.1 mM EDTA, 2.5% IGEPAL) with protease inhibitor cocktail (Sigma). After centrifugation, supernatant was discarded and the pellet was resuspended in 150 μl of Buffer B (20 mM HEPES, (pH 7.9), 0.4 M NaCl, 1 mM EDTA, 10% glycerol) with protease inhibitor cocktail. After shaking for 2 h at 4°C, the sample was centrifuged and the supernatant (nuclear extract) was collected and stored at -80°C.

Nuclear extract from liver of rats (Fischer 344) was prepared as described previously [20]. Briefly, liver homogenate was prepared in 4 volumes (w/v) of ice-cold buffer (0.25 M sucrose, 15 mM Tris-HCl (pH 7.9), 16 mM KCl, 15 mM NaCl, 5 mM EDTA, 1 mM EGTA, 1 mM DTT, 0.15 mM spermine, 0.15 mM spermidine; with 0.1 mM PMSF, 2 μg/ml leupeptin and 5 μg/ml aprotinin) and centrifuged for 10 min at 2000 × g. To the pellets 4 volumes of ice-cold buffer (10 mM HEPES; pH 7.9, 1.5 mM MgCl_2_, 10 mM KCl and protease inhibitors) was added. After gentle resuspension of the pellet centrifugation was carried out for 10 min at 4000 × g. The pelleted nuclei was resuspended in ice cold buffer (10 mM HEPES (pH 7.9), 0.75 mM MgCl_2_, 0.5 M KCl, 0.5 mM EDTA, 12.5% glycerol and protease inhibitors) and incubated on ice for 30 min with continuous agitation. The mixture was centrifuged for 30 min at 14,000 × g and supernatants (nuclear extracts) were collected.

### Cloning of the 5’-flanking region of human *PPA1* gene

The genomic DNA was isolated from MCF7 cells as per standard protocol [21]. A 1217 bp of 5′-flanking region of the *PPA1* gene, from—1143 to +74 bp relative to the translation start site (designated as +1) was searched from Genbank and amplified by PCR using primers Forward (-1035 Luc F) having Kpn-I restriction site and Reverse (+74 Luc R) having Xho-I restriction site and genomic DNA from MCF7 cell line was used as template. The PCR amplification was carried out for 35 cycles using step cycle of 94°C for 30 s, 57°C for 40 s, 72°C for 1.30 min followed by final extension at 72°C for 10 min with 1 unit of Platinum taq polymerase (Invitrogen). The PCR product was purified using Qiaquick Gel extraction kit (Qiagen,USA). Both PCR product and pGL3-basic vector were digested with Kpn-I and Xho-I restriction enzymes (Fermentas). The digested fragment was then ligated to restriction enzyme digested pGL3-basic vector using DNA ligase and designated as pGL3-1217.

### Generation of 5’-serially deleted *PPA1* promoter fragments and its cloning into pGL3-Basic vector

The 5’-serially deleted *PPA1* promoter constructs were generated by PCR using pGL3-1217 construct as template. All the primers used in the generation of deletion constructs were listed in Table 1. The 5’-serially deleted promoter constructs were cloned into pGL3-Basic vector and designated as pGL3-1143 (-1143/-1), pGL3-530 (-530/-1), pGL3-266(-266/-1), pGL3-187(-187/-1) and pGL3-137(-137/-1).

**Table 1:** Primers used for promoter amplification

Primers	Sequences (5’→3’)
-1035 Luc F	ACAGGTACCGAAATCCTGGCCATGAAAT
-530 Luc F	ACAGGTACCTGTTACGACGGATCAGAAA
-266 Luc F	ACAGGTACCTCTGAGCCCTAGCTGCCATC
-187 Luc F	ACAGGTACCGTGCGCCTGCGCACGGGGTT
-137 Luc F	ACAGGTACCCCGCGTTAAAGGCGCTCCCC
-1 Luc R	ACACTCGAGAGTGCCGGAGTCCTGCCGC
+74 Luc R	ACACTCGAGAGACACTCACTGAGGAAGACT

### Electrophoretic mobility shift assays (EMSAs)

Electrophoretic mobility shift assays were carried out as described previously [22] with minor modifications. Oligonucleotides corresponding to predicted Sp1 binding sites were synthesized as shown in Table 2. The 5’-end labelling of oligonucleotides with [γ-^32^P] ATP were carried out using T4 polynucleotide kinase (Promega). These 5’-end labelled oligonucleotides were annealed to the corresponding complementary oligonucleotides to form double stranded oligonucleotides. Nuclear extract was incubated with the labelled DNA probe in the binding buffer mix (10 mM Tris, pH 7.5, 1 mM EDTA, 6% glycerol, 1 mM DTT, 0.3 mM PMSF, 5 μg BSA and 1.0 μg poly (dI-dC)) for 20 min at room temperature. In the competitive EMSA experiments 100–200 fold molar excess of unlabelled Sp1 consensus oligonucleotides were added as cold competitors to the reaction mixture 10 min prior to the addition of labelled probe. Reaction mixtures were separated on a 6% non-denaturing polyacrylamide gel, dried and autoradiographed.

**Table 2:** Oligonucleotides used in EMSA.

Oligos	Sequences (5’→3’)
PPA1 Sp1 site1 F	GGCGCTCCCCGCCCCGCCCGCCGG
PPA1 Sp1 site1 R	CCGGCGGGCGGGGCGGGGAGCGCC
PPA1 Sp1 site2 F	GGCCCGGCGGGGGTGGGAACACT
PPA1 Sp1 site2 R	AGTGTTCCCACCCCCGCCGGGCC
PPA1 Sp1 site3 F	CCCGAGGGGCGGGGCTGGGGAGTGC
PPA1 Sp1 site3 R	GCACTCCCCAGCCCCGCCCCTCGGG

### DNase I footprinting

DNase I footprinting was carried out as described [23]. Briefly, end-labeled rat *Ppa1* proximal promoter fragment of 276 bp (-338 to -63 bp relative to the translational start site) was prepared by PCR using following primers: 5'-GAATAACTAGCGCTCTGTCCCG-3’ and 5’-CAGCTTCGGAGTGGTTGTACC-3’ and rat *Ppa1* promoter plasmid [24] as template (Genbank accession no: DQ978330). End-labelled promoter fragment (50 fmoles) was incubated with 50 μg of rat liver nuclear extract and 2 μg of poly (dI-dC) in binding buffer (10 mM Tris-HCl (pH 7.5), 50 mM NaCl, 1 mM EDTA, 1 mM DTT and 5% glycerol) at room temperature for 30 min. Then 7.5 mM MgCl_2_ and 5 mM CaCl_2_ were added to the reaction mixture and incubated with DNase I (0.25 U, Roche, USA) for 60 s at room temperature. Control experiments were set without nuclear extracts and digested with 10-fold less DNase I. Digestion was stopped by adding an equal volume of stop solution (1% SDS, 20 mM EDTA, 400 mM NaCl, 100 μg/ml yeast tRNA and 200 μg/ml proteinase K) and samples were incubated at 45°C for 60 min. Then phenol/chloroform extraction and ethanol precipitation was carried out. DNase I digested sample and sequencing reaction products were electrophoresed on 6% polyacrylamide sequencing and autoradiographed.

### ChIP assay

Chromatin immunoprecipitation in MCF7 cells was carried out as described previously [25] with minor modifications. Briefly, MCF7 cells were cross linked with a final concentration of 1% (V/V) formaldehyde (Sigma) for 10 min at room temperature. Cells were washed twice with cold PBS and lysed in 1 ml of lysis buffer (1% SDS, 10 mM EDTA, 50 mM Tris-HCl, pH 8.1) for 10 min on ice. Cell lysate was sonicated to shear the DNA to fragment lengths between 200 bp to 1 kb in size. The lysate was centrifuged for 10 min at 14,000 rpm at 4°C and sonicated extract was diluted 10 fold in ChIP dilution buffer (0.01% SDS, 1.1% Triton X-100, 1.2 mM EDTA, 16.7 mM Tris-HCl (pH 8.1), 167 mM NaCl). The diluted extract was precleared at 4°C with sepharose beads (GE Healthcare) for 1 h. Aliquots of the lysate were incubated overnight with 2.0 μg of antibody at 4°C with rotation. The antibody-protein complex was precipitated by blocked protein A agarose beads and the pelleted beads were washed with Wash Buffers (once with Low Salt Buffer, High Salt Buffer, LiCl Buffer and twice with TE Buffer). The immune complexes were eluted in 1% SDS/ 0.1 M NaHCO_3_ and reverse crosslinked for 6 h at 65°C. Following proteinase-K (Sigma) treatment, the samples were extracted with phenol-chloroform and precipitated with ethanol. The recovered DNA was dissolved in TE Buffer and used for PCR amplification. Antibodies used were: Sp1 antibody (sc-14027 X, Santa Cruz), p300 antibody (sc-584, Santa Cruz) and Rabbit IgG (kch-504-250, Diagenode).

### Site-directed mutagenesis

Mutagenesis of putative transcription factor binding sites on *PPA1* promoter was accomplished by a three step PCR procedure. The first PCR reaction was carried with a 5’-primer containing a Kpn-I restriction site and the antisense primer containing mutated nucleotides. The second PCR reaction was carried out by using a 3’-primer containing a Xho-I restriction site and a sense primer complementary to its antisense primer. In the two PCR reactions described above, the pGL3-266 plasmid was used as a template. PCR fragments were purified using Qiagen Kit and used as the templates for the third PCR reaction with wild type 5’ and 3’ primers. The fragments with mutated transcription factor binding sites were cloned into Kpn-I and Xho-I restriction sites of pGL3-Basic vector. All the mutated constructs were verified by sequencing.

All the primers used in the procedure are listed in the Table 3.

**Table 3:** Primers used for site-directed mutagenesis.

Primers	Sequences (5’→3’)*
PPA1 mutSp1 site1 F	GGCGCTCCCCGC**AAA**GCCCGCC
PPA1 mutSp1 site1 R	GGCGGGC**TTT**GCGGGGAGCGCC
PPA1 mutSp1 site2 F	GGCCCGGCGG**TA**GTGG**C**AACACT
PPA1 mutSp1 site2 R	AGTGTT**G**CCAC**TA**CCGCCGGGCC
PPA1 mutSp1 site3 F	CCCGAGG**TT**CGGG**T**CTGGGGAGTGC
PPA1 mutSp1 site3 R	GCACTCCCCAG**A**CCCG**AA**CCTCGGG

*The bold and underlined letters denote the mutated sequences.

### Transfection and Luciferase assay

MCF7 cells were transiently transfected using GeneCellin HTC transfection reagent (Biocellchallenge, France) according to manufacturer’s instructions. pRL-TK vector (Promega), a Renilla luciferase expressing vector was used as an internal control in transfections to normalize transfection efficiency. In Sp1 or p300 transfected and TSA treated samples, relative luciferase activity was normalized to total protein content in the samples due to fluctuations in renilla luciferase activity. Cells were analyzed for luciferase activity using Dual-Luciferase Reporter Assay System (Promega) according to manufacturer’s instructions and luciferase activity was measured using a luminometer (SIRIUS). All the transfections were carried out in triplicates in three independent experiments. The statistical analyses were carried out by one-way ANOVA using Graph Pad Prism 5.01 software. Unpaired t-test was carried out for comparison between two groups. P values less than 0.05 were considered statistically significant.

### Whole cell lysate preparation and western blot analysis

Whole cell lysates were prepared from cell lines using RIPA buffer and western blotting was carried out as described previously [26]. Images were acquired by Chemidoc XRS+ molecular imager (Bio-Rad, USA). Antibodies used were: Sp1 antibody (sc-14027, Santacruz), Anti-PPA1 (WH0005464M1, Sigma) and Monoclonal Anti-α-Tubulin antibody (T5168, Sigma).

### Chromatin accessibility assay

Nuclei from MCF7 cells were isolated as described earlier [27]. Chromatin accessibility assay was carried out by incubating 20 μg of chromatin with 3 Kunitz Unit of Micrococcal Nuclease (Neb). Reactions were carried out at 37° C for 12 min in a water bath and stopped by adding Stop buffer (100 mM EDTA and 10 mM EGTA) and 5 μl of 20% SDS. Purified DNA was analyzed by semi-quantitative PCR using *PPA1* promoter primers spanning the proximal Sp1 sites. Actin promoter was amplified from each sample as representative control using following primers: 5’-CAGCACCCCAAGGCGGCCAACGC-3’ and 5’-GCAACTTTCGGAACGGCGCACGC-3’ [28].

### Colony Formation Assay

MCF7 cells were transfected either with PPA1 shRNA (Sigma) or scramble shRNA and selected with 1μg/ml puromycin (Gibco, A11138-03). Scramble shRNA was a gift from David Sabatini (Addgene plasmid # 1864) [29]. For colony formation assay, the cells were seeded in 12-well plate and kept in puromycin containing medium for two weeks. The colonies were fixed with methanol:acetic acid mixture (3:1 ratio) and stained with 0.1% crystal violet (Acros Organics, USA) and counted manually.

### MTT Assay

For cell viability assay, shRNA transfected MCF7 cells were splitted into 24 well plate at a density of 2x10^5^ cells per well. Next day, the medium was changed to fresh medium containing puromycin (1 μg/ml). Cell viabilities were determined by MTT assay after 24 and 48 h of puromycin treatment. For MTT assay, 25 μl of 5 mg/ml MTT (MP Biomedicals) solution was added to each well and incubated for 4 h at 37°C. The formazan crystals were dissolved in DMSO and analyzed using a microplate reader (Varioskan Flash, Thermo Scientific, USA) at 570 nm with background subtraction at 630 nm.

## Results

### Primer Extension analysis

**Fig 1:**
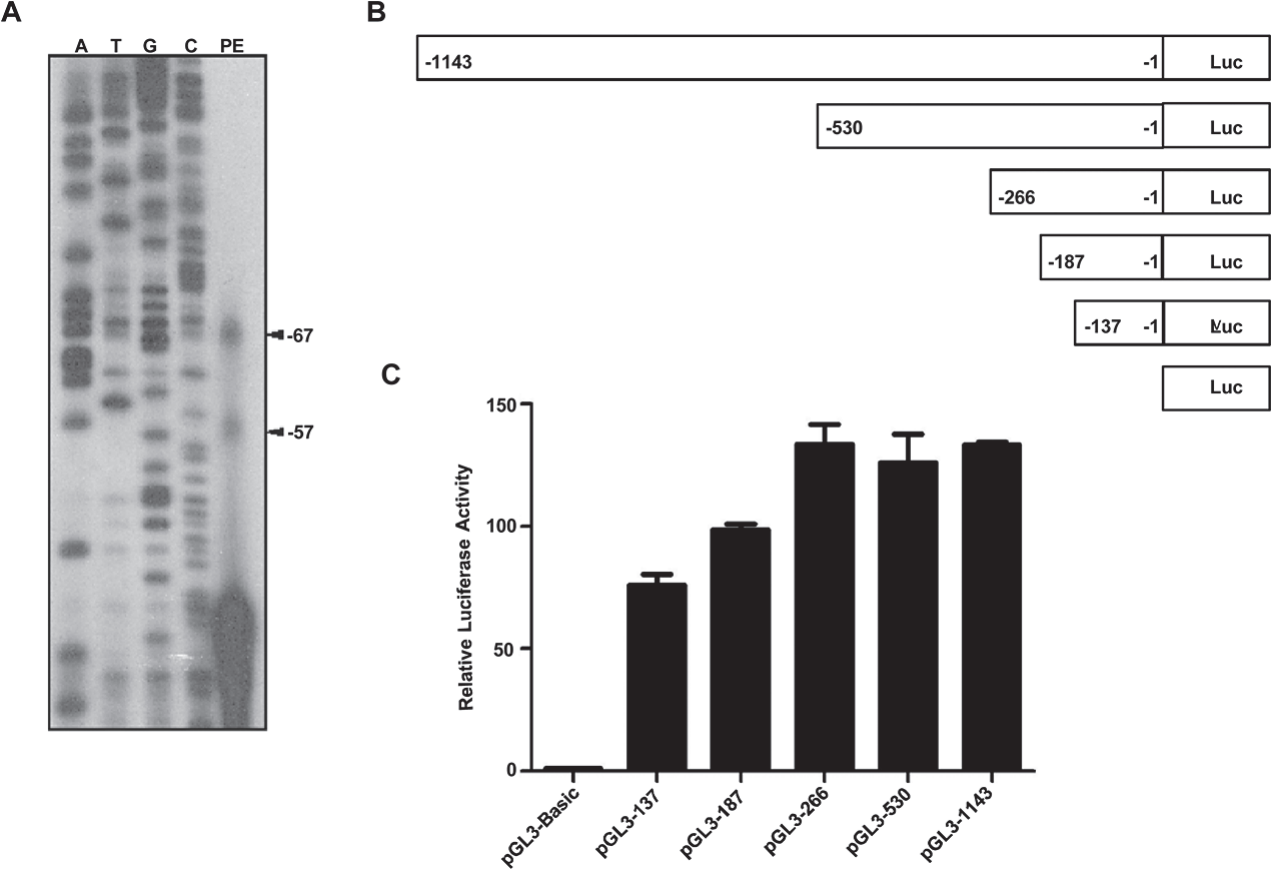
Identification of *PPA1* minimal promoter. (A) Determination of transcription start site of *PPA1* gene. Total RNA was isolated from MCF7 cells and analyzed by primer extension analysis using a primer complementary to the -20 to -1 region of exon 1 of *PPA1* gene. The sequencing reaction was set using same primer as in primer extension. Both the sequencing reaction products (A, T, G and C) and primer extension product (PE) were run on a 7.5% urea-polyacrylamide gel. (B) Schematic representation of 5’-deletion constructs of *PPA1* promoter. All the deletion constructs were generated by PCR using pGL3-1217 construct as template and cloned into the Kpn-I and Xho-I restriction site of pGL3-Basic vector. (C) Promoter activity of all the 5’-deleted promoter constructs was determined by Dual-Luciferase assay. MCF7 cells were transfected with promoter constructs and after 24 h luciferase assay was carried out using Dual-Luciferase assay kit (Promega). Each transfection was conducted in triplicate and the results are represented as mean ± S.E.M.

Primer extension analysis was carried out using a primer PPA1PER which is complementary to the -20 to -1 region of exon 1 of *PPA1* gene. As shown in (Fig 1A), two transcription start sites at 67 bp and 57 bp away from translation start site were found. TATA-less promoters are often known to be associated with multiple transcription start sites [30].

### Cloning of the 5’-flanking region and identification of the *PPA1* minimal promoter region

To understand the mechanism of transcriptional regulation of the *PPA1* gene, a 1217 bp of its 5’-flanking region (-1143/+74 from the first translation start site ATG, designated as +1) was amplified by PCR and cloned in a pGL3 basic vector. Next, to identify the minimal promoter region, 5’-deletion constructs were generated and cloned into pGL3 basic vector and named as pGL3-1143, pGL3-530, pGL3-266, pGL3-187 and pGL3-137 (Fig 1B). These deleted constructs were transfected in MCF7 cells and dual luciferase assay was carried out. On comparison it was found that the construct pGL3-266 is sufficient to drive most of the luciferase activity (Fig 1C). No significant alterations of luciferase activity were observed beyond -266. Hence, it suggests that the fragment between—266 to -1 may contain important regulatory elements.

### Confirmation of binding of Sp1 through EMSA and ChIP assay

**Fig 2:**
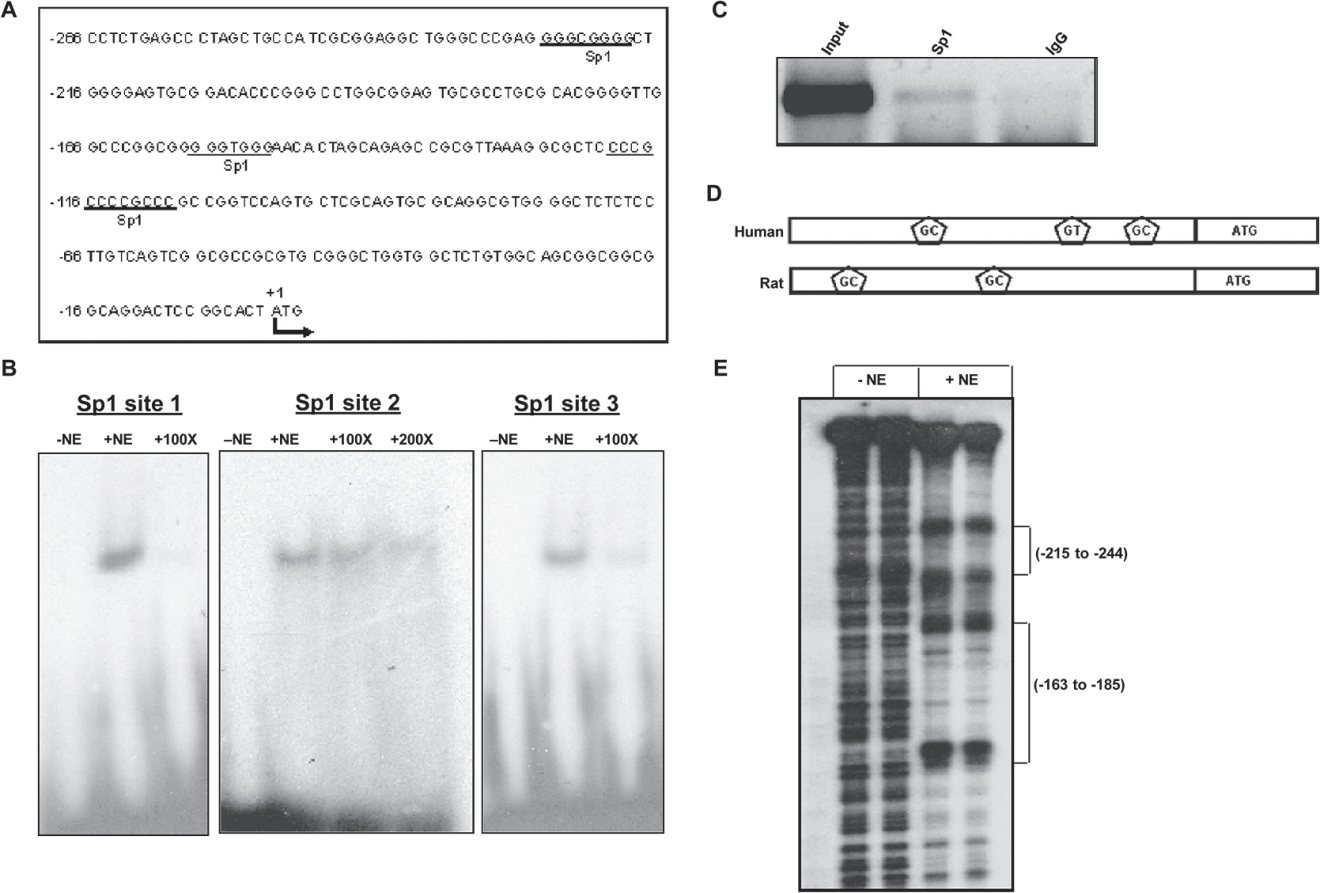
Interaction of Sp1 with *PPA1* promoter. (A) Schematic presentation of predicted Sp1 binding sites on the *PPA1* core promoter region. (B) Electrophoretic Mobility Shift Assay (EMSA) for the three predicted Sp1 sites on *PPA1* promoter. Radiolabelled oligonucleotides corresponding to the predicted Sp1 sites were incubated with MCF7 cell nuclear extract. In competitive experiments 100–200 fold molar excess of unlabeled consensus Sp1 oligonucleotides were added.—NE lane denotes no nuclear extract added, +NE lane denotes addition of MCF7 cell nuclear extract and 100x and 200x lanes explains the reactions in which either 100 fold or 200 fold molar excess of cold consensus oligonucleotides were added along with the nuclear extract. (C) Chromatin Immunoprecipitation assay showing the interaction of Sp1 with the *PPA1* promoter inside the MCF7 cells. (D) Diagrammatic representation of Sp1 binding sites on *PPA1* promoter of human and *Ppa1* promoter of rat from the translational start site (ATG) of the respective genes. (E) DNase I protected regions were identified over the two Sp1 binding sites on the *Ppa1* promoter of rat.

To find out the important *cis* elements, the promoter region sequences between -266/-1 were analyzed. As shown in Fig 2A, two putative Sp1 binding sites (GC-boxes) were found by TFSEARCH (http://www.cbrc.jp/research/db/TFSEARCH.html) and one GT-box, known to be the binding sites for Sp1, was found on *PPA1* promoter. To confirm the binding of Sp1 with the putative response elements EMSAs were carried out. In EMSA experiments specific complexes were formed by radiolabelled double-stranded oligonucleotides (site 1 (-127 to -104), site 2 (-167 to -145) and site 3 (-232 to -208)) and MCF7 nuclear extracts. The complex formation was abolished by competition with 100–200 fold molar excess of unlabeled consensus oligonucleotide duplexes for Sp1 (Fig 2B).

To confirm the binding of Sp1 to the *PPA1* promoter in vivo, a chip assay was carried out in MCF7 cells using antibodies specific to Sp1. The proximal promoter fragment including the three Sp1 sites was amplified by PCR from the immunoprecipitated DNA. Thus, the Sp1 binds to the *PPA1* promoter in vivo (Fig 2C).

Similar to human *PPA1* promoter, presence of two Sp1 binding sites on the rat *cytosolic inorganic pyrophosphatase* (*Ppa1*) promoter (Fig 2D) and interactions of Sp1 to these sites was reported previously through EMSA experiments [24]. We further characterize this interaction of Sp1 with the rat *Ppa1* promoter by DNase I footprinting analysis and footprints (-185 to -163 and -244 to -215) were identified over the Sp1 binding sites (Fig 2E). Presence of Sp1 on the human and rat *inorganic pyrophosphatase* promoter suggests that Sp1 may be an important transcription factor in the regulation of gene expression.

### Sp1 transactivates *PPA1* promoter constructs and upregulates protein expression

**Fig 3:**
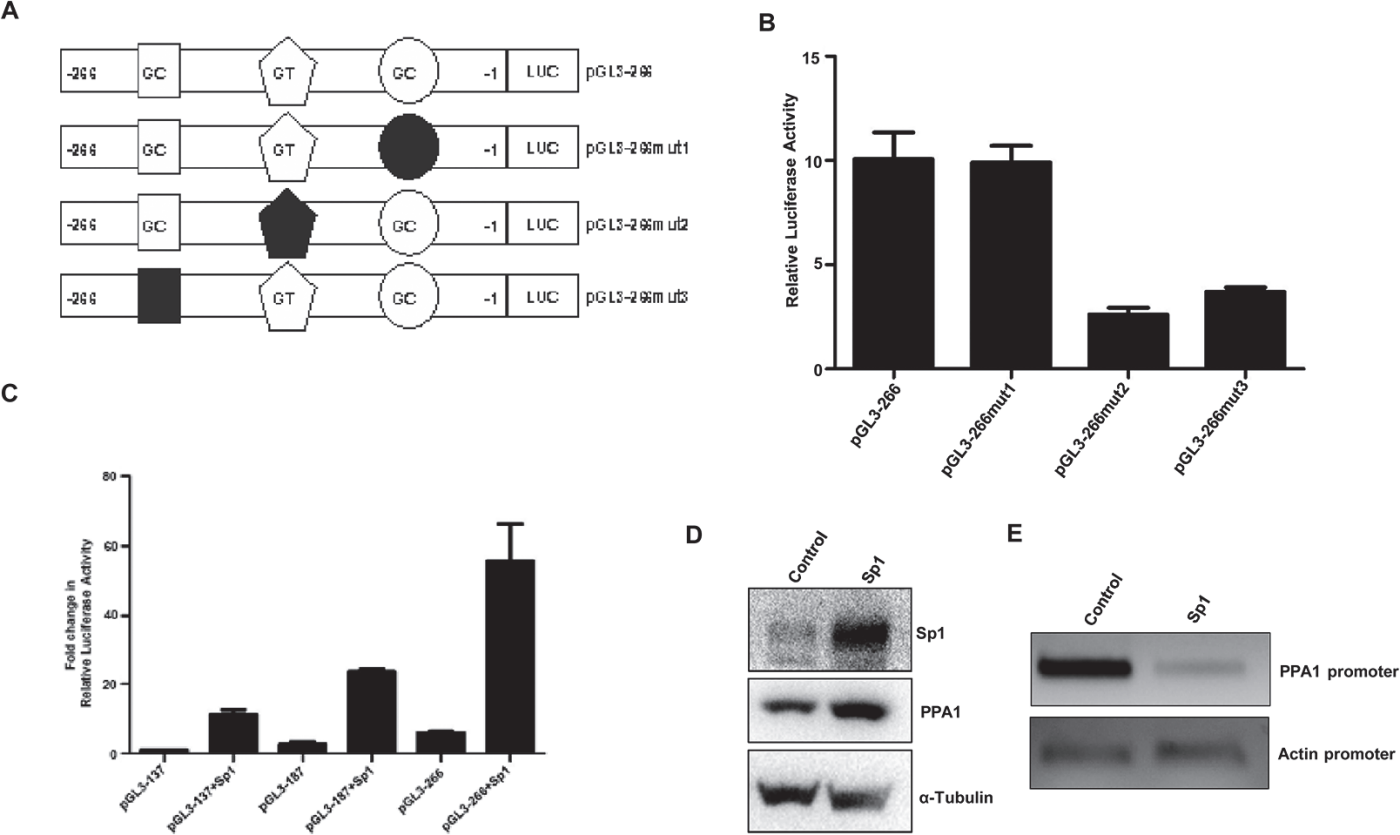
Sp1 regulates PPA1 expression and alters chromatin accessibility. (A) Schematic representation of Sp1 binding site mutated promoter constructs generated by site-directed mutagenesis. All the mutated promoter constructs were generated by a three step PCR method and cloned into pGL3-Basic vector. (B) Luciferase assay of mutated promoter constructs was carried out using Dual-Luciferase assay kit (Promega). (C) Luciferase assay showing the effect of Sp1 overexpression on the promoter deletion constructs. pGL3-137, pGL3-187 and pGL3-266 have one, two and three Sp1 binding sites respectively. Each transfection was carried in triplicate and the results represent mean ± S.E.M of two independent experiments. (D) Overexpression of Sp1 led to the increased expression of PPA1. MCF7 cells were transiently transfected with empty vector (control), or Sp1 expression vector. After 48 h of transfection, cells lysates were prepared for western blot experiment. (E) Sp1 alters chromatin accessibility across *PPA1* promoter. Nuclei from MCF7 cells transfected with empty vector (control) or Sp1 expression vector were subjected to MNase digestion and then the purified DNA was amplified by semi-quantitative PCR using primers for the proximal promoter region of *PPA1*. β-Actin promoter region was amplified as a representative control.

To identify which of these Sp1 binding sites are important in the *PPA1* gene regulation, pGL3-266 mutant constructs for each of the Sp1 binding sites were generated by site-directed mutagenesis (Fig 3A). Mutations in the Sp1 binding site 2 (-167/-145) and site 3 (-232/-208) resulted approximately 74% and 63% decrease in luciferase activity respectively compared to the wild type promoter construct (Fig 3B). Mutating site 1 (-127/-104) did not significantly affects promoter activity.

To study the role of Sp1 in the up regulation of *PPA1* promoter, Sp1 expression vector was cotransfected with *PPA1* promoter reporter constructs (pGL3-266, pGL3-187 and pGL3-137) and luciferase assay was carried out. Sp1 expression vector (pcDNA3.1-Sp1-V5) was kindly provided by Professor Ya Wen Lin (Graduate Institute of Medical Sciences, National Defense Medical Center, Taipei, Taiwan). Overexpression of Sp1 increased the promoter activities of all the constructs, but construct pGL3-266 having three Sp1 sites, had highest activity upon Sp1 expression vector cotransfection than pGL3-187 and pGL3-137 (Fig 3C). Also, the PPA1 protein expression was upregulated by Sp1 overexpression (Fig 3D). These data suggest that, all the three Sp1 sites are responsive to Sp1 overexpression and *PPA1* promoter activity and protein expression can be up regulated by Sp1.

### Overexpression of Sp1 alters the *PPA1* chromatin accessibility

The differential expression of a gene at specific environment depends on the accessibility of transcription factor to the chromosomal DNA. PPA1 is deregulated in several cancers and differentially expressed in rat and mice liver with age. In order to address the role of Sp1 in chromatin accessibility, nuclei from Sp1 transfected cells were isolated and treated with Micrococcal Nuclease (MNase). Then genomic DNA was isolated and subjected to PCR amplification using primers spanning proximal region having Sp1 sites. An increase in MNase accessibility was observed across the *PPA1* promoter with Sp1 overexpression but there was no change in the accessibility of the control β-actin promoter (Fig 3E). Hence, it suggests that Sp1 may be altering the chromatin accessibility at the *PPA1* promoter.

### p300 transactivates *PPA1* promoter and stimulates Sp1 dependent activation of transcription in MCF7 cells

**Fig 4:**
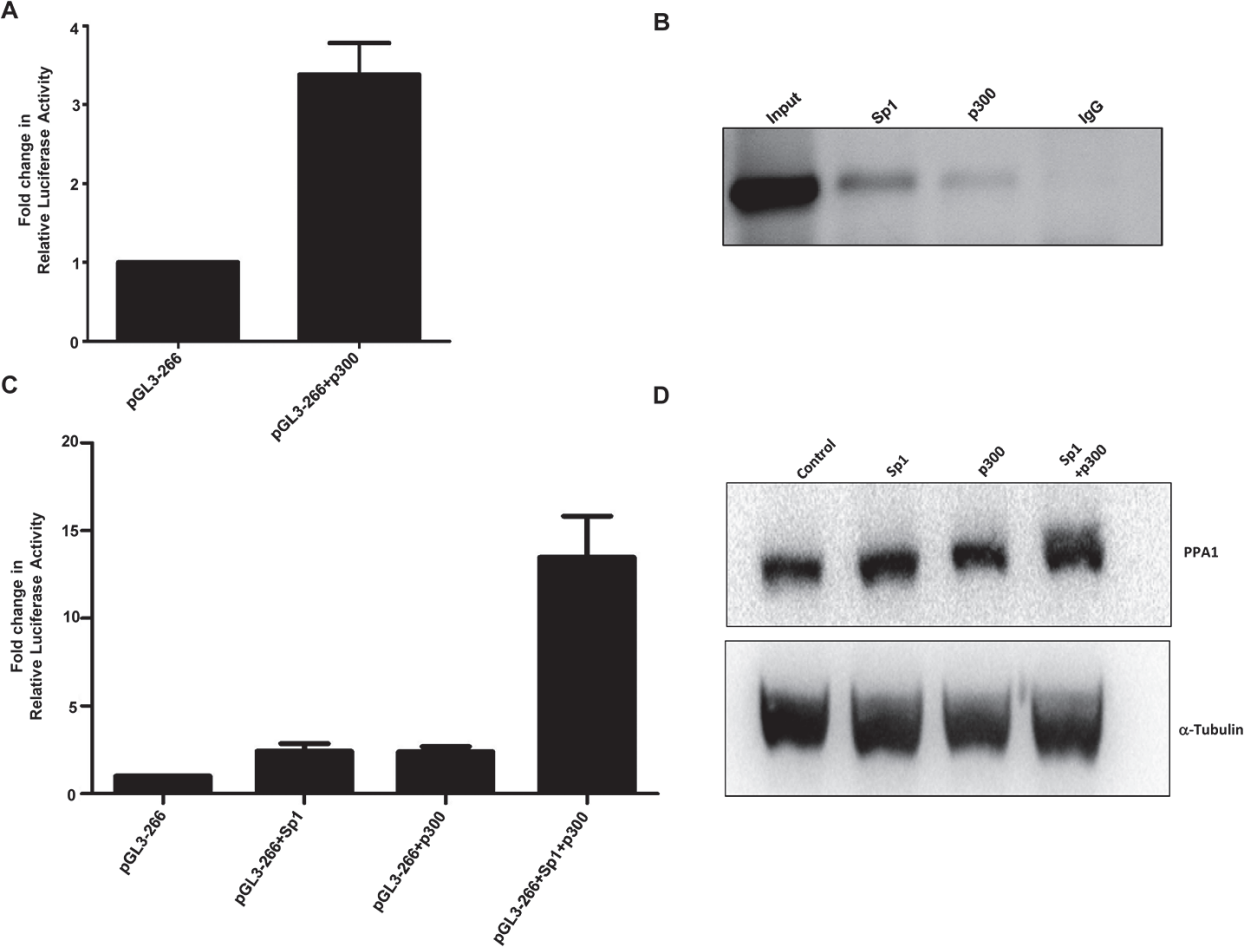
p300 stimulates Sp1 mediated transactivation of *PPA1* promoter. (A) Overexpression of p300 upregulates *PPA1* promoter activity in MCF7 cells. The results are represented as mean ± S.E.M of three independent experiments. (B) p300 associates with *PPA1* promoter. ChIP assay was carried out in MCF7 cells and presence of p300 on the *PPA1* proximal promoter region to which Sp1 also binds was confirmed. (C) p300 and Sp1 activates *PPA1* promoter cooperatively. MCF7 cells were transfected with different plasmids as illustrated and luciferase assay was carried out as described previously. The results represent mean ± S.E.M of two independent experiments. (D) Western blot showing the expression of PPA1 with the overexpression of Sp1 and p300.

Since it is observed that Sp1 increases the chromatin accessibility at the promoter region of *PPA1* gene, we hypothesized that there may be some coactivator which is playing a role in the transcriptional regulation of the gene. It is earlier reported that Sp1 with the cooperation of p300 regulate the expression of certain genes [31–33]. Hence, we next studied the possible involvement of p300 in the transcriptional regulation of *PPA1* gene. The p300 wild-type expression plasmid pCI-p300 was kindly provided by Dr. Joan Boyes (Institute of Cancer Research, London, UK). As shown in Fig 4A, upon overexpression, p300 was able to transactivate the *PPA1* promoter activity. Also, binding of p300 to the *PPA1* promoter was studied by ChIP assay and the presence of p300 was found at the proximal promoter region (Fig 4B). Next, we studied the effect of p300 on the Sp1 induced activation of *PPA1* expression by overexpressing p300 and Sp1 in MCF7 cells. As shown in Fig 4C and 4D, p300 acts as a coactivator by further stimulating Sp1-mediated transactivation of *PPA1* promoter activity and expression.

### TSA (HDAC inhibitor) and HAT activity of p300 affects *PPA1* expression and promoter activity

**Fig 5:**
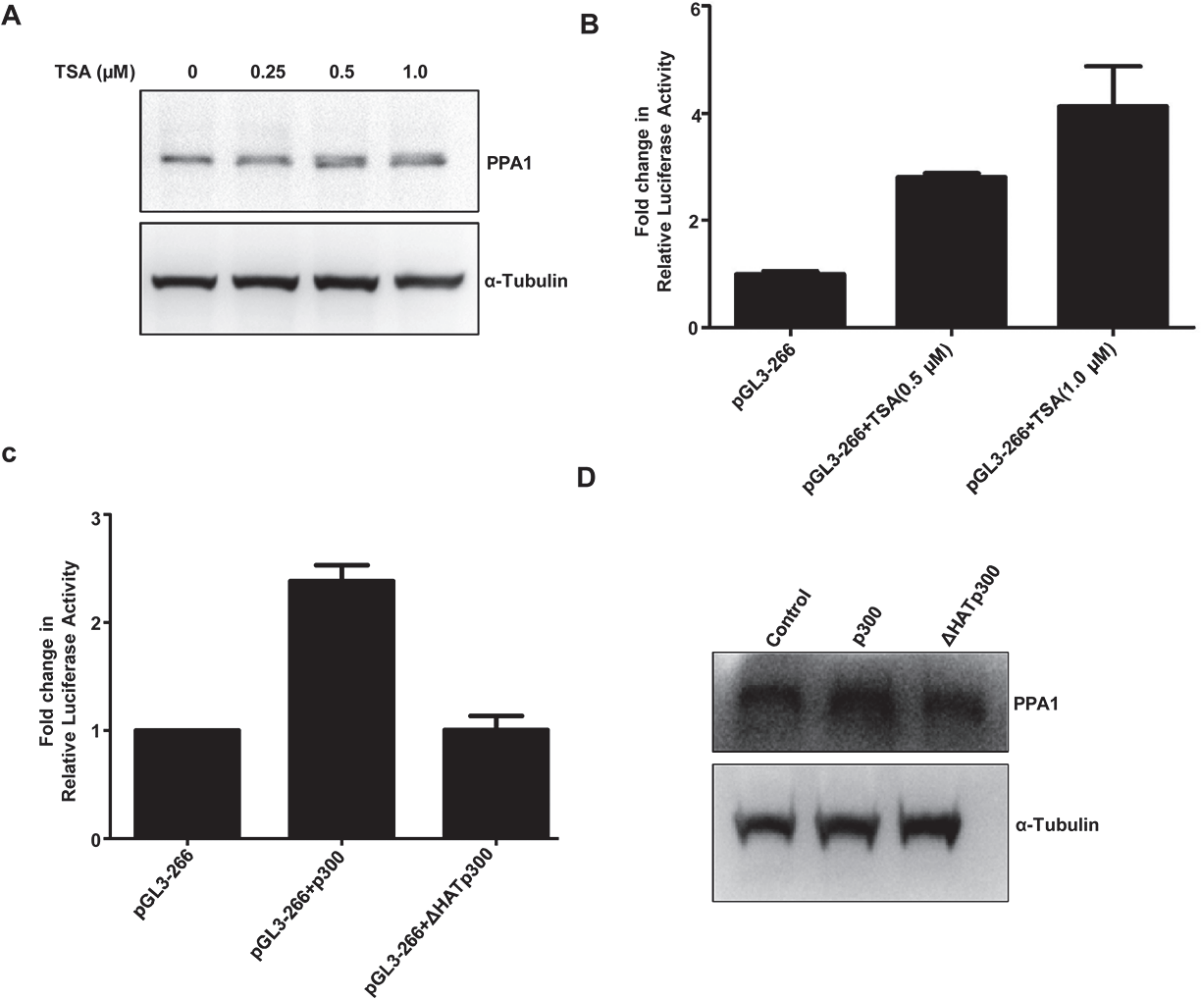
Effect of TSA and HAT activity of p300 on *PPA1* expression and promoter activity. (A) Western blot showing the expression of PPA1 in MCF7 cells treated with 0.25, 0.5 and 1 μM TSA for 24 h. (B) TSA treatment increases *PPA1* promoter activity in MCF7 cells. Each transfection was conducted in triplicate and the results are represented as mean ± S.E.M. (C) Luciferase assay showing the *PPA1* promoter activity with the overexpression of p300 and ΔHATp300. The results represent mean ± S.E.M of three independent experiments. (D) Representative western blot showing the expression level of PPA1 with the overexpression of p300 and ΔHATp300. Western blotting was carried out using the lysates obtained in previous luciferase assay experiment.

Since p300, which also has intrinsic acetyl transferase activity, increased *PPA1* promoter activity and expression, we assume that histone acetylation modification may have role in PPA1 regulation. To understand whether *PPA1* gene is regulated epigenetically via histone acetylation/deacetylation, MCF7 cells were treated with various doses of TSA, a histone deacetylase inhibitor for 24 h and western blot analysis was carried out. PPA1 protein expression was found to be upregulated in TSA treated samples (Fig 5A). Next, the effect of TSA on *PPA1* promoter was studied to confirm its transcriptional regulation of *PPA1* gene. Luc-266 promoter construct was transfected in MCF7 cells and after 12 h of transfection cells were treated with 0.5 μM and 1 μM TSA for 24 h. As shown in Fig 5B, a significant increase in *PPA1* promoter activity was found upon TSA treatment.

To determine the importance of HAT activity of p300, a mutant p300 expression vector lacking HAT domain (1472–1522), ΔHATp300 was cotransfected with the PPA1 promoter construct in MCF7 cells and luciferase assay was carried out. ΔHATp300 expression vector was a kind gift from Dr. Joan Boyes (Institute of Cancer Research, London, UK). As shown in Fig 5C and 5D, *PPA1* promoter activity and expression was not upregulated significantly with the overexpression of HAT deleted as compared to wild type p300. Hence, the above results suggested that the HAT activity of p300 is involved in P300 mediated *PPA1* regulation.

### Knockdown of *PPA1* decreases colony formation and cell viability in MCF7 cells

**Fig 6:**
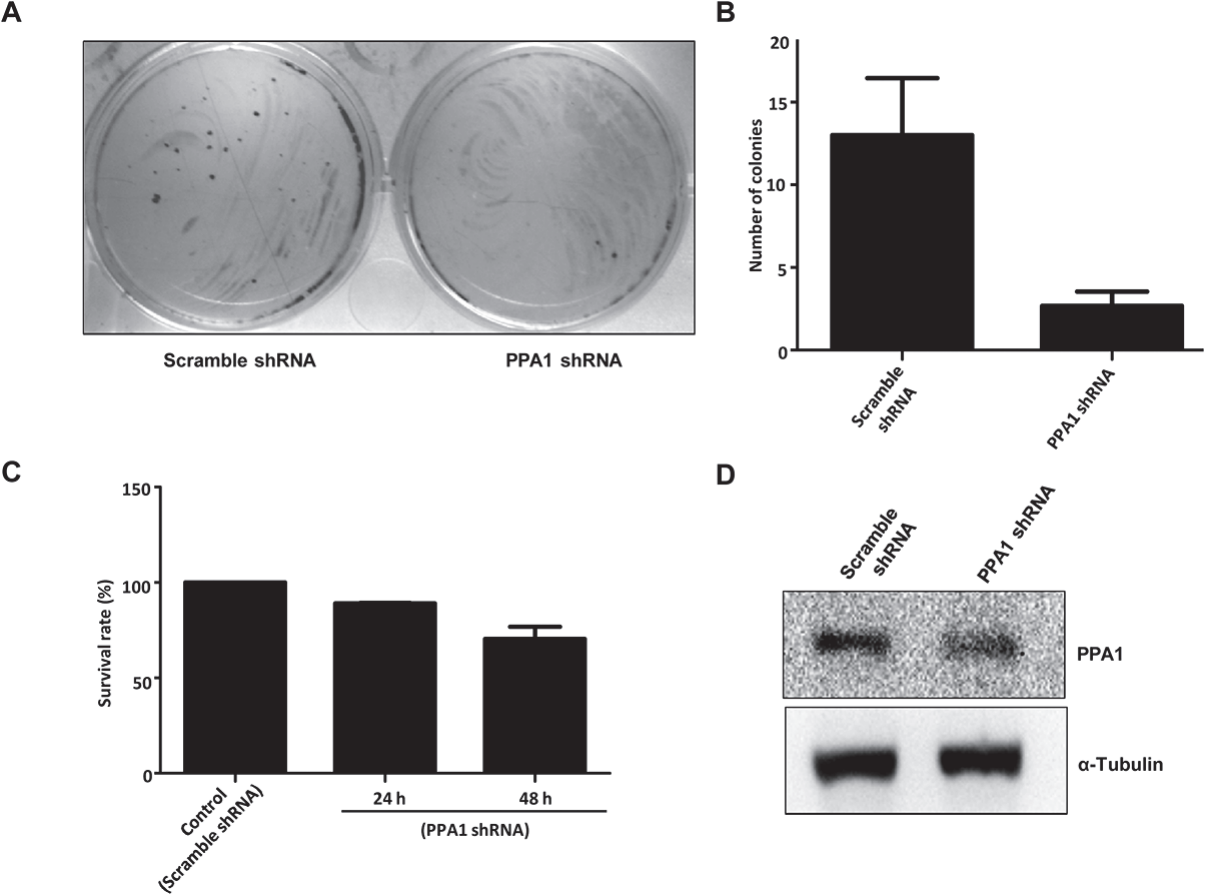
Effect of *PPA1* knockdown on the colony formation and cell viability in MCF7 cells. (A) Representative picture of colony formation assay in MCF7 cells. PPA1 shRNA and scramble shRNA transfected cells were seeded in 12 well plates and colonies were visualized by crystal violet staining after 2 weeks of puromycin selection. (B) Number of colonies of MCF7 cells transfected with scramble shRNA or, PPA1 shRNA from triplicate wells, p˂0.05. (C) Effect of PPA1 shRNA on the cell survival. MCF7 cells transfected with scramble shRNA or PPA1 shRNA were seeded in 24 well plates in triplicate and selected with puromycin. Cell viability was analyzed by the MTT assay at 24 h and 48 h of puromycin selection. Data represents mean ± S.E.M of two independent experiments, p˂0.05. (D) Western blot showing knockdown of PPA1 after 48 h of transfection of PPA1 shRNA in MCF7 cells.

To study the role of PPA1 knockdown in MCF7 cell growth or survival, colony formation assay was carried out. As shown in Fig 6A, knockdown of *PPA1* significantly decreased the colony forming ability compared to control cells. Further, down regulation of *PPA1* on cell viability was studied by MTT assay and as shown in Fig 6C, knockdown of *PPA1* significantly reduced cell viability compared to control cells after 48 h of puromycin selection. Results of both the experiments were in agreement with each other.

## Discussion

Gene expression is involved in the regulation of the fundamental cellular processes [34] and deregulated transcription is linked to several human diseases [35, 36]. Cancer is one of such diseases in which aberrant gene expression plays a critical role in its progression. Identification and characterization of the deregulated genes will be helpful in understanding the mechanism behind the cancer progression and improvement of diagnosis and treatment [37].

Inorganic pyrophosphatase (PPA1), an important pyrophosphate hydrolysing enzyme is also known to be overexpressed in many types of cancer. In gastric cancer cells, PPA1 plays a role in migration and invasion [17] and in ovarian cancer cells silencing of PPA1 inhibits the proliferation [18].

In the present study, we have identified and studied the functional promoter region of the *PPA1* gene using MCF7 cell line (the human breast cancer cell line) as a model. The 5’-flanking region of the *PPA1* gene was cloned and the promoter fragment -266 to -1 was identified to be the minimal promoter region. This promoter region was analyzed for the presence of important regulatory elements and three putative Sp1 binding sites were identified. Binding of Sp1 to this promoter fragment was confirmed by EMSA and ChIP assays. Importance of the identified Sp1 binding sites was studied by site-directed mutagenesis and Dual-Luciferase assay. Mutation of binding site 2 and 3 decreased the promoter activity by 74% and 63% respectively, as compared to the wild type promoter construct and Sp1 could be an important transcription factor involved in the regulation of *PPA1*.

Sp1 is a ubiquitously expressed transcription factor which belongs to the Kruppel-like Zinc-finger transcription factor family [38]. Sp1 binds to GC/GT boxes on the promoters [39, 40] and regulates expression of many genes [41]. Sp1 is known to be overexpressed or overactivated in many types of cancers and plays a role in carcinogenesis. Sp1 is overexpressed in gastric cancer [42], in breast cancer [43], in thyroid tumors [44] and in pancreatic adenocarcinoma, Sp1 overexpression is associated with higher grade and lymph node metastasis [45]. In MCF7 breast cancer cells Sp1 is involved in the basal and estrogen-induced gene expression and cell cycle progression [46] and in gastric tumor cells knock down of Sp1 expression suppressed angiogenesis [47]. Sp1 also regulates cancer cell metabolism by regulating a number of metabolic genes in cancers [48]. In-addition several post-translational modifications in Sp1 affecting its transcriptional activity play a role in tumorigenesis [49].

To study the role of Sp1 in regulating *PPA1* transcription, *PPA1* promoter constructs were cotransfected with Sp1 expression vector in MCF7 cells and upon luciferase assay *PPA1* promoter activity was found to be increased by Sp1. Also, the overexpression of Sp1 increased the PPA1 protein level and chromatin accessibility in MCF7 cells. Chromatin remodeling is an important mechanism involved in various cellular processes (gene expression, apoptosis, DNA repair) and dysregulation of these processes can lead to cancer [50, 51]. Previous studies have shown that Sp1 mediates chromatin remodeling by recruiting HATs or HDACs to the promoters [52]. Therefore, in order to find out whether any coactivator is involved in the *PPA1* regulation, we studied the role of p300, a member of histone acetyltransferases family. Presence of p300 on the *PPA1* promoter region, which contains Sp1 transcription factor binding sites, was confirmed by ChIP assay and overexpression of p300 was found to upregulate the *PPA1* promoter activity and also potentiated the Sp1-mediated transactivation of *PPA1* gene.

Further, to find out the effect of histone acetylation on the regulation of *PPA1* gene expression, TSA—a specific inhibitor of HDACs was used. Both the PPA1 protein expression and promoter activity was enhanced significantly in MCF7 cells. Also, the HAT activity of p300 was found to be important in p300 mediated transactivation of *PPA1* promoter and expression. HAT deleted p300 did not transactivate the *PPA1* promoter significantly as compared to the wild type p300.

Histone acetylation and deacetylation is a dynamic process across the promoter region which plays important role in the gene regulation. Various HATs and HDACs are recruited to the specific region to carry out histone acetylation/deacetylation and the coordination between these events is critical for maintenance of unique gene expression. Deregulated histone acetylation/deacetylation is associated with various diseases [53]. We also evaluated the effect of *PPA1* knockdown on the growth/survival of MCF7 cells by colony formation assay and MTT assay. Knockdown of *PPA1* decreased the colony forming ability and survival of MCF7 cells.

Further in depth studies will elucidate more on the *PPA1* gene regulation in cancer and its role in carcinogenesis.
